# Perceived Organizational Support for Enhancing Welfare at Work: A Regression Tree Model

**DOI:** 10.3389/fpsyg.2016.01770

**Published:** 2016-12-27

**Authors:** Gabriele Giorgi, David Dubin, Javier Fiz Perez

**Affiliations:** ^1^Department of Psychology, European University of RomeRome, Italy; ^2^Psychological ARTSAustin, TX, USA

**Keywords:** perceived organizational support, work-related stress, welfare, health promotion, workplace, organizational psychology

## Abstract

When trying to examine outcomes such as welfare and well-being, research tends to focus on main effects and take into account limited numbers of variables at a time. There are a number of techniques that may help address this problem. For example, many statistical packages available in R provide easy-to-use methods of modeling complicated analysis such as classification and tree regression (i.e., recursive partitioning). The present research illustrates the value of recursive partitioning in the prediction of perceived organizational support in a sample of more than 6000 Italian bankers. Utilizing the tree function party package in R, we estimated a regression tree model predicting perceived organizational support from a multitude of job characteristics including job demand, lack of job control, lack of supervisor support, training, etc. The resulting model appears particularly helpful in pointing out several interactions in the prediction of perceived organizational support. In particular, training is the dominant factor. Another dimension that seems to influence organizational support is reporting (perceived communication about safety and stress concerns). Results are discussed from a theoretical and methodological point of view.

## Introduction

Employee welfare is an “umbrella concept” including various services, benefits, and facilities offered to employees with the aim of fostering their working conditions and professional growth. Assessments of welfare measures are both objective and subjective. The former includes interventions regarding allowances, housing, transportation, medical insurance, wellness coverage, and so on (Schmitz and Schrader, 2015). The latter includes less tangible benefits such as perceived working conditions, the interpersonal environment in which work takes place, and organizational actions and support perceived by employees (Zhong et al., 2016).

Popular discourse suggests employees working under stressful conditions or experiencing problems in life-balance are increasing. As a result, it is important to examine factors that can foster personal well-being and professional growth with a potential return of investment in terms of higher productivity and greater loyalty. Indeed, employees who perceive that their organization is supportive show higher performance, proactive behaviors, reduced absenteeism, and a lessened intention of quitting their job (Eisenberger et al., 1997; Riggle et al., 2009; Arshadi and Hayavi, 2013; Caesens et al., 2016).

Such an analysis is particularly important given that in some countries (e.g., Southern-Europe) the economic crisis is still ongoing with negative consequences for salaries and job stability (Mucci et al., 2016). In Italy, for instance, salaries have not increased significantly in the last 7 years, and salaries for public employees have remained frozen (Italian National Institute of Statistic—Istat, 2015). Consequently, as renumeration cannot be the first driver of employee motivation, non-economical reward is increasing with the aim of fostering perceived organizational support (see for instance the inter-ministries decree, 25 march, 2016 that introduce favorable taxation for welfare activities, Ministro del Lavoro e delle Politiche Sociali and Ministro dell'Economia e delle Finanze, 2016). Consequently, under economic turbulence, organizational support is crucial for developing employees' productivity and achieving business success (Choi and Lin, 2009).

The goal of the current paper is to present a recursive partitioning analysis in order to examine combinations of working conditions and organizational variables that contribute to employees' perceptions of organizational support.

### Perceived organizational support and health

Perceived organizational support (POS) is defined as the employees' “*beliefs concerning the extent to which the organization values their contribution and cares about their well-being*” (Eisenberger et al., 1986, p. 501).

Organizational support literature theorizes that a factor of business success is the extent employees develop beliefs concerning organization orientation to employees' welfare such as the organization valuing employees' contributions and caring about their well-being (Eisenberger and Stinglhamber, 2011). On one hand, employees develop an organization's identification evaluating the received treatment in the workplace. This perception is important both for business success and employee health (Eisenberger et al., 2002). On the other hand, social exchange theorists argue that receiving increased welfare activities from their organizations might contribute more to success. Specifically, employees compensate their employer with higher work performance (Eisenberger et al., 1986).

In a systematic review of 70 studies, Rhoades and Eisenberger (2002) found that positive organizational support is related to fair organizational procedures, supervisor support, and favorable rewards and job conditions, which in turn lead to positive outcomes for both the individual and the organization, such as increased affective commitment to the organization, increased performance, and reduced withdrawal behaviors.

In particular, Riggle et al. (2009) conducted an important meta-analytic study including 167 works. Results indicated that POS had a strong association with job satisfaction, organizational commitment, and intention to leave; whereas only moderate, positive effect on employee performance.

Research has pointed out that work organizations can be regarded as “employees' perceptions” as they represent contexts where people tend to assign the organization humanlike characteristics (Eisenberger et al., 1986) considering the company having its own unique capability, just like people do. Indeed POS seems characterized by specific psychological processes: the reciprocity norm, —the employee's felt obligation to care about the organization's welfare and to help the organization reach its mission—, the fulfillment of socio-emotional needs—leading employees to identify positively in the organization—, the development of beliefs that the organization recognizes and rewards increased performance (i.e., performance-reward expectancies; Rhoades and Eisenberger, 2002; Eisenberger et al., 2014; Kurtessis et al., 2015).

In the first process, when employees trust their organization they develop a sense of obligation. They might work harder as well as increasing voluntary citizenship behaviors because the basis of social exchange is the norm of reciprocity (Eisenberger et al., 1986, 2001; Rhoades and Eisenberger, 2002).

In the second process, employees consider their organization a source of social and emotional resources and are dedicated to work because they feel valued, cared for, and esteemed. As a consequence emotional identification toward the organization (brand, costumers) may increase with a higher levels of teamwork satisfaction and an increased perception of the positive image of the company (Eisenberger et al., 1990; Edwards, 2009; Edwards and Peccei, 2010).

Finally, the belief that one is being rewarded fairly in the organization motivates employees to work well and engage in activities without tangible rewards (e.g., extra role behaviors) (Rhoades and Eisenberger, 2002).

An increasingly important aspect central to the psychological process of the perceived organizational support theory is the perceived consideration of employee well-being and welfare.

Accordingly, a systematic review on POS conducted by Baran et al. (2012) analyzed 249 studies and found that the primary theoretical theme in association with POS was employee well-being (*n* = 43).

In particular, employees who experience lower levels of welfare and wellbeing might deteriorate the reciprocation psychological process when they don't feel organizationally and emotionally supported (e.g., when employees experience organizational stress).

Accordingly, social exchange theory points out that POS is negatively associated with stress (Baran et al., 2012). For instance companies who care about their employees' well-being are more likely to improve working conditions and job design, such as reducing conflicting job requirements (Jawahar et al., 2007) or bullying and incivility in workplace (Miner et al., 2012).

Additionally, POS, fulfilling socio-emotional needs, increase employees organizational membership, and role status (Rhoades and Eisenberger, 2002) and this plays a central role in developing a healthy employee–employer relationship and in reducing stress. POS fulfills emotional needs, decreases strain and thus enhances well-being (Byrne and Hochwarter, 2006).

Furthermore, several researches found an association between POS with health-related variables such as sense of accomplishment (Jain and Sinha, 2005), organization-based self-esteem (Lee and Peccei, 2007), general health (Bradley and Cartwright, 2002), decreased burnout (Kang et al., 2010), and anger (O'Neill et al., 2009).

As far as POS antecedents is concerned, job conditions (such as aspects of training, job discretion, role stressors, relations in workplace), seem to have a impact. These job characteristics overlap with the concept of stress, especially if we consider recent expanded models that go beyond job demand and job control based models (see for instance Giorgi et al., 2013). Similarly, individual antecedent such as personality factors and socio-demographic variables seemed to have a weaker relationship with POS (Baran et al., 2012).

Our theoretical framework is based on the proposition that POS is associated with job characteristics of the working environment such as stress and welfare related factors.

### POS organizational antecedents

Recently, a further systematic study and meta-analysis were conducted on POS pointing out its antecedents. Findings of the Ishfaq and Muhammad' (2015) review work, which included 170 studies, revealed that POS was mainly linked to justice, growth opportunities, supervisor support, and coworkers support.

Kurtessis et al. (2015) performed a meta-analysis, which included 492 papers containing 558 studies, grouped antecedents of POS into three categories: (a) treatment by organization members, (b) employee–organization relationship quality, (c) HR practices and job conditions.

In particular, job conditions played a substantial role in establishing POS, with employees being inclined to perceive organizational support as a way to reciprocate adequate working conditions (Shore et al., 2006; Kurtessis et al., 2015).

In addition, Kurtessis et al.'s study posited that POS is connected to favorable social interactions, both with colleagues and supervisors, with the latter more significant because leaders are perceived as representative of the organizations (Stinglhamber and Vandenberghe, 2003). Finally, contextual factors that convey the organization's regard for employees were associated with POS: common values shared with employee, psychological contracts, fairness of treatment, and perceived organizational politics (e.g., Eisenberger et al., 2001) An organizational factor not extensively investigated by the literature in connection with POS is training.

Conversely, high organizational concern with developing employees is linked with health and business success and might stimulate in workers a general felt obligation to reciprocate toward the organization in positive manner (Eisenberger et al., 1990; Kurtessis et al., 2015).

Instead, research has more recently looked at the relationship between low or high level of POS with safety perception. Safety climate seemed to play an important role in POS (Wallace et al., 2006). Similarly, as noted by Mearns and Reader (2008), the reporting of errors of unsafe situations was associated positively POS. On the other hand, the association of POS with safety seemed to be influenced by further variables (job demand, leadership, etc.) as previously noted.

Kath et al. study (2010) suggested that safety perception, as other stress related working conditions, have likely important associations with POS but do not represent exclusive antecedents (Kurtessis et al., 2015). To sum up, various stressors and strains need to be investigated in order to delineate more clearly the combination of interrelated processes that may occur in POS.

Accordingly, although meta-analysis exists in the field of POS (Kurtessis et al., 2015), current research offers limited empirical evidence of the work-related mechanisms and psychological processes potentially interacting in relation to perceived organizational support.

Given that multiple dimensions and distinguished categories have been suggested in studying POS, we expect specific combinations of antecedents (e.g., working conditions, social support, and work pressure) that interact with one another to impact worker's perceptions. This is also in accordance with theorists who advocate that the investigations of concurrent interconnected organizational variables for the prediction of several outcomes (Scott et al., 2011).

In doing so, we use regression tree analysis to explore main perceived organizational support relationships with correlated organizational and stress factors.

### Recursive partitioning models and applications in the health field

Of the parametric methods used in occupational health, regression is the most common. Regression carries with it a laundry list of requirements including linearity, normality, independence of error terms, and constant variance of error terms that need to be met in order to perform well (i.e., have unbiased parameter estimates). However, it is often the case in health research and practice that these conditions are not met or is overly restrictive. A particular limitation associated with parametric models is that it is challenging to estimate and interpret interactions occurring amongst more than two variables or to model the impact of variables with non-linear functional forms (Strobl et al., 2009a).

Yet, when trying to examine outcomes such as well-being, research tends to focus on main effects, and take into account limited numbers of variables at a time.

Regression tree models are based on graphs in which each circle in the diagram reflects a binary splitting point (i.e., a splitting node) in the model. Specifically, the application of graphical models on work related stress is motivated by the possibility to describe graphically the multitude of relations and dependencies among different variables. Recursive partitioning has been used in a variety of different fields to explain drug treatment retention (Hellemann et al., 2009) survival of cancer patients (Grossman and Ram, 2014); treatment effects in clinical trials (Doove et al., 2014); clusters in genomics (Nilsen et al., 2013); intimate partner violence (Salis et al., 2014); and to help detect problem gamblers (Markham et al., 2013), among others.

The present research illustrates the value of recursive partitioning in the prediction of perceived organizational support in a sample of more than 6000 Italian bankers.

## Materials and methods

### Procedure and participants

Data were collected by a team of researchers with the aim of measuring work-related stress and perceived organizational support. The survey was designed in accordance with privacy and anonymity regulations and was administered on-line through the company intranet. In particular, employees were informed of the purpose of the assessment and of the aggregate data analysis performed by qualified academic researchers. Information of data protection were presented in a precise manner, so the test-takers' security was enhanced.

The target population was an Italian national bank with multiple locations widespread in Italy. All the employees were invited to fill in the questionnaire on the bank intranet portal.

The participation for the study was considerable for on-line survey, as around 30% of employees filled in the questionnaire. Questionnaires with more than four missing items were deleted from the database in order to increase the validity of the research. The final sample consisted of 6588 bankers (of which 57.5% were males). In addition, 4.3% of the respondents were <30 years old, 26.5% were 31–40 years old, 38.7% were 41–50 years, 28.7%, were between 51 and 60 years old; 1.7% of the respondents were over 60 years old. Regarding job seniority, 9.7% of the respondents had worked for up to 7 years in their current company; 25.1% for 8–15 years, 42.3% for, 16–30 years 22.8% for more than 30 years. Finally, 91% were office workers whereas 9% were managers or middle managers who co-ordinated a team.

### Measures

The Stress Questionnaire (SQ) was chosen for this investigation because it is a validated Italian tool that measures not only work-related stress but also organizational perceptions. In brief, SQ has multiple scales which investigate classical stressors as well as emergent stressors and contextual variables. All the utilized scales are part of the SQ and detailed information can be found in the test validation study (Giorgi et al., 2013). The “**Psychosocial risk scale of the Stress Questionnaire**” (Giorgi et al., 2013) consists of 25 items and 5 subscales: job demand, lack of job control, role conflict, lack of supervisors' support, and lack of colleagues' support. A 5 point Likert scale ranging from 1 (absolutely agree) to 5 (absolutely disagree) was used: (a) role conflict, which measures the perception of lack of awareness in their roles and responsibilities (5 items: e.g., “I have a clear idea about what is expected of me at work,” reverse scored) Alpha.84; (b) lack of colleagues' support or collaboration and support among employees (5 items: e.g., “I get the support I need from colleagues,” reverse score it's difficult to assess whether my colleagues are competent”) Alpha.79; (c) lack of supervisors' support or the extent to which employees don't experience support and understanding from their supervisors/leaders (5 items: e.g., “My supervisor energizes me at work,” reverse scored “My supervisor is neither competent nor self-confident”) Alpha.82; (d) job demands, which refers to quantitative, demanding aspects of the job (6 items: e.g., “I have unrealistic deadlines,” “I'm under pressure at work”), Alpha.81; and e) lack job control or job resources that pertain to the task (5 items: e.g., “I can plan my work,” reverse scored “I'm fully autonomous in choosing my working tools” reverse scored) Alpha.77. The factorial structure and the reliability of this scale have been supported in previous studies (Giorgi et al., 2013).

The scale “**Perceived organizational support**” comprises 4 items in a 5-point Likert-scale (from 1: “strongly disagree” to 5: “strongly agree”) and measures the extent to which the organization values and cares for employees' welfare (e.g., “This organization pays little attention to the interests and well-being of its employees” reverse coded—; “This organization tries to take care of its employees' welfare”), Alpha.82. Consequently, after computing a total mean score, a higher score refers to higher perceived organizational support. This scale was included in the Stress Questionnaire (Giorgi et al., 2013).

The scale “**Traning**” consist of 3 items in a 5-points Likert-scale (from 1: “strongly disagree” to 5: “strongly agree”) and measures the perceived quality of training in workplace (e.g., “This organization trains adequately employees for performing job tasks” reverse scored; “employees are not well trained when they have to use a new tool/equipment”), Alpha.74.

Higher scores mean that employees perceived a lack of training or low quality training. This scale is part of the Stress Questionnaire (Giorgi et al., 2013).

The scale “**Reporting**” measures the employees' perception of an unsafe and unhealthy environment wherein procedures of safety and health are invisible or discouraged/under-reported (“workers are not trained neither informed about job related risks; “I can freely report stress and safety risks perceptions—for me and for others” (reverse coded) Alpha.52. It was measured using a four-item scale of the Stress Questionnaire (Giorgi et al., 2013).

### Analysis

Descriptives, Alpha of Conbrach, and correlation were calculated.

Regression Tree analysis was used beginning with a generation of a root node. After, a splitting rule based on algorithm, determined cutoff points that minimize the within-group variance on the outcomes. Progressively further binary splits were performed in order to divide the sample into subsamples, called nodes that ended finally in terminal nodes at the bottom of the figure.

## Results

Descriptive statistics and correlations are displayed in Table 1. As expected, Stress questionnaire variables were related to POS.

**Table 1:** **Descriptive statistics and correlations**.

**Variables**	***M***	***SD***	**1**	**2**	**3**	**4**	**5**	**6**	**7**	**8**
1. Job demand	3.21	0.75	(0.81)							
2. Lack of job control	2.86	0.71	0.51^**^	(0.77)						
3. Role conflict	2.13	0.68	0.10^**^	0.37^**^	(0.84)					
4. Lack of supervisors support	2.60	0.85	0.25^**^	0.34^**^	0.34^**^	(0.82)				
5. Lack of colleagues support	2.48	0.69	0.34^**^	0.38^**^	0.29^**^	0.51^**^	(0.79)			
6. Training	3.35	0.80	0.33^**^	0.37^**^	0.31^**^	0.35^**^	0.32^**^	(0.74)		
7. POS	2.51	0.77	−0.42^**^	−0.46^**^	−0.30^**^	−0.43^**^	−0.38^**^	−0.63^**^	(0.82)	
8. Reporting	2.98	0.61	0.36^**^	0.43^**^	0.33^**^	0.36^**^	0.38^**^	0.49^**^	0.54^**^	(0.52)

*Internal Consistency coefficients (Cronbach's alphas, Cronbach, 1951) appear along the diagonal in parentheses*.

** *p < 0.01*.

**Figure 1:**
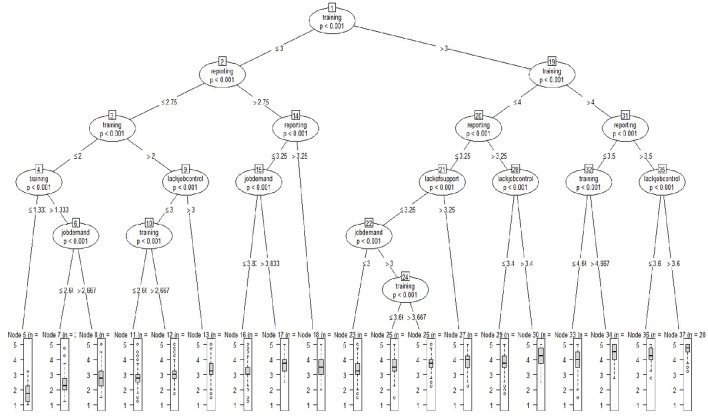
**Regression tree predicting perceived organizational support**. All variables in tree are positively coded.

Utilizing the ctree function party package in R (Hothorn et al., 2015; see also Strobl et al., 2009b), we estimated a regression tree model predicting perceived organizational support from the Stress Questionnaire. The optimal tree identified is displayed in Figure 1.

The ctree function performs recursive partitioning using a conditional inference framework developed by Strasser and Weber (1999). For this study, recursive partitioning was carried out by first testing the global null hypothesis of independence between any of the job characteristics and POS. Based on this initial analysis, it was determined that several dependencies between POS and the job characteristics existed and, of these, splitting the training variable into two groups (above and below 2.667) had the strongest association to POS. The strength of the association is measured by the *p*-value corresponding to a test for the partial null hypothesis of the training variable and POS. Because this binary split on the training variable holds the strongest association with POS it appears at the top of the tree (i.e., node 1). Continuing on from node 1, the tree splits and recursively repeats the first step; i.e., searching for the strongest association between the remaining job characteristics or other splits of the variable that has already been split—in this case the training variable. Following the tree diagram in Figure 1 to node 2, the largest association occurs with the same variable (i.e., training), but this time at a binary split of 1.667. At this location of the tree, the data is again split into those employees below 1.667 on the training variable and those above 1.667 but <2.667, the split on node 1. On the other side of the tree at node 21, the strongest association with POS for those employees who perceive a high degree of training (i.e., >2.667) is with the variable of reporting split into groups at a score of 3. The analysis ends when no additional associations meet the criteria for a meaningful association. In this study, the criterion was set where an association must have a *p* < 0.000000001 in order to avoid capitalizing on effects due to chance. The bottom of the figure displays the terminal nodes and the distribution of perceived organizational support for individuals falling into each of the categories.

Overall, the figure illustrates a number of “pathways” to perceived organizational support. For example, particularly high levels of perceived organizational support occur amongst those who strongly feel they can report stress and safety risk perceptions (reporting) and among those workers who perceive they are trained (training; node 37). High levels of perceived organizational support also occur when individuals have lower levels of training (compared to node 37) but perceive more job control (node 32). POS is lowest amongst those who have low training, reporting, and job control (node 5). Note that variables not in the tree do not contribute significantly to the purity (i.e., homogeneity) of categories.

It's worthwhile to note that training is the primary splitter at the root node (Node 1) and appears again in several root nodes (Node 2, 7, 17, 27, 30, 33) demonstrating that, the extent of perceived training in workplace, generates different profiles. Reporting also appears multiple times within the tree (Node 3, 10, 21, 22).

For those who report medium levels of supervisor support and reporting (Node 14 and 22), job demand further explains differences in overall level of perceived organizational support.

Finally, multiple pathways to perceived organizational support are evident in the terminal nodes, with the contribution of the majority of the tested variables.

Further results are found by following the pathways in the figure below.

## Discussion and conclusions

The field of organizational psychology has amassed numerous predictors of key outcomes (e.g., job performance, well-being), yet our standard regression methods prohibit modeling a large number of these predictors simultaneously, testing them against each other or, perhaps more importantly, examining how they function in conjunction.

It is precisely these challenges for which non-parametric data mining techniques are suited. In particular, techniques such as recursive partitioning can be fruitfully applied to high dimensionality problems (i.e., small n, large p), complex data structures, and problems with many predictor variables (Strobl et al., 2009b). These methods have the potential to greatly improve prediction.

In this study, we examined a diverse set of organizational variables, using the novel approach of Regression Tree, which identified combinations among predictors of POS. In particular we considered supportive welfare practices in the wellbeing/stress area relevant to determining employee POS.

Study aims linked to a recent meta-analytic literature emphasizing the simultaneous examination and the interactive nature of the causes of POS. It also resonates well with the stress literature, as it demonstrates that significant organizational stressors rarely occur in isolation, but might generate interactive or combined effects (Ward, 2014).

As shown, the regression tree model contains five organizational variables (training, reporting, lack of job control, lack of support, and job demand), that sorted the sample into 19 groups through their interactions. Where a number of POS antecedents are present at the same time, synergetic effects seemed to occur.

In our study, the most dominant contributing factor on POS was training. This finding is consistent with the study of Eisenberger et al. (1997), and the recent met-analysis of Kurtessis et al. (2015) which indicated that “*training (developmental) opportunity was the job condition most strongly viewed as under the discretionary control of organizations and, presumably, most indicative of POS*.” p. 11.

In addition, employees may simply have higher POS or become more attached to the organization because being well trained fulfills their socio-emotional needs (Di Fabio, 2014, 2015). Alternatively, consistent with organizational support theory, good training practice might be associated with POS because they are likely to be viewed as discretionary rather than compulsory and this aspect is theoretically fundamental for POS.

Training may also be considered a factor of protection from work instabilities that seems very widespread in the Italian context. This might be a promoting factor of POS (Di Fabio and Saklofske, 2014; Di Fabio and Kenny, 2015). For example, training practices might be perceived visible manifestations of invisible organizational characteristics (such as economical power). However, this issue should be investigated further.

Job control that considers resources, such as autonomy and discretionarily, confirmed its impact on POS in our study. Similarly, we pointed out the importance of supervisors' support on POS, and this finding is consistent with prior works (Shoss et al., 2013). Many studies described POS related to the leadership style and supervisory practices that play a key role in providing growth opportunities and organizational benefits to employees (Eisenberger and Stinglhamber, 2011).

In our study, tree analysis excluded colleagues' support and role ambiguity/role conflict, because they were not significant contributors to the model in comparison to the variables left in the tree diagram. Indeed, supervisors support has been highlighted as more important than colleagues's support (Kurtessis et al., 2015). Similarly, the impact of role conflict on determining POS is lower than intuitively expected. Eisenberger et al. (1997) pointed out that role stressors are less perceived to be under organizational control with respect to job enrichment elements and consequently marginally related to POS. Similarly, in our study the effect of job demand factors was limited and found only in the final nodes. According to Eisenberger and Stinglhamber (2011), job pressure is particularly due to the job characteristics rather than to the perceptions of employers and organizational support.

While our study confirms the results of studies examining POS organizational antecedents, this research is one of the few to examine the factor called “reporting” in association with POS. People might be inclined to reciprocate less, or disengage because they can't feel secure about their own health and safety.

Our findings show that reporting in combination with other organizational variables might create lesser POS because such negative perceptions of health and safety conditions generate a greater concern for the individual's well-being (Rousseau and Aubé, 2010; Caricati et al., 2016). In addition, the role of reporting in the model seems particularly important as it interacts with the dominant factor training as well as with further elements. This result partially follows the literature, as the relationship of safety communication with POS was non-exclusive but related to several organizational factors such as leader-member exchange, job demands, etc. (Kath et al., 2010).

This study however presents some limitations that should be considered while interpreting our findings. A first limitation relates to the use of self-report measures, raising questions about common method bias. A second limitation is the lack of a longitudinal design. Hence, this study should be replicated with specific temporal design in order to fully understand how organizational perceptions lead to others. A third limitation is that the sample is not representative of the Italian bankers and was limited to a single organization. Further, the present study was conducted in an Italian setting. Socio-economical differences may impact POS (Eisenberger et al., 1986) across organizations and countries (Baran et al., 2012). The Italian banking sector, which is encountering a turbulent economical time (Quaglia and Royo, 2015), appears at risk of developing lesser POS.

Finally, contextual factors such as value congruence, (psychological contract and fairness), were not investigated, whereas in literature a strong association with POS was found (Cropanzano et al., 2001; Cropanzano and Mitchell, 2005).

In conclusion the factor “reporting” is new in the literature and would benefit from refinement. The reliability of the scale in this study appeared also limited.

However, our study provides new research knowledge. The major strengths are the large sample of bankers (a difficult population to sample in psychology), the use of a new statistical method such as the regression tree model, and the fact that the study responds to the call of Italian regulation for welfare promotion.

Further, the study replies to a call of the literature to investigate (a) the association of POS with wellbeing, (b) the construct of POS in non-U.S. samples, (c) the measurement of POS in contexts with job instabilities. In addition, it is essential to emphasize that these findings can be used effectively to generate theoretical developments and organizational interventions. By having determined the interaction of antecedents that are predictive of POS, specific interventions can be implemented rather than be broadly focused.

The perceived organizational support experienced by employees as a result of a combination of working conditions (such as training and reporting) may cause important implications for generating a favorable orientation toward the organization stimulating welfare activities engagement. Indeed, as explained in the introduction, in Italy the government is promoting concrete welfare activities, however the choice of using these bonuses stand not only in the employer, but also in the employees and consequently, their perception of POS, might play a key role. These assumptions suggest the need for additional research to further refine the role of POS in the employee–organization relationship for practical and scientific purposes.

## Author contributions

All authors listed, have made substantial, direct, and intellectual contribution to the work, and approved it for publication.

### Conflict of interest statement

The authors declare that the research was conducted in the absence of any commercial or financial relationships that could be construed as a potential conflict of interest.
