# Corrigendum: Perceived Organizational Support for Enhancing Welfare at Work: A Regression Tree Model

**DOI:** 10.3389/fpsyg.2017.00331

**Published:** 2017-03-03

**Authors:** Gabriele Giorgi, David Dubin, Javier Fiz Perez

**Affiliations:** ^1^Department of Psychology, European University of RomeRome, Italy; ^2^Psychological ARTSAustin, TX, USA

**Keywords:** perceived organizational support, work-related stress, welfare, health promotion, workplace, organizational psychology

In the original article, the information in the published Figure 1 was too small to read. The corrected figure appears below. The authors apologize for this error and state that this does not change the scientific conclusions of the article in any way.

**Figure 1:**
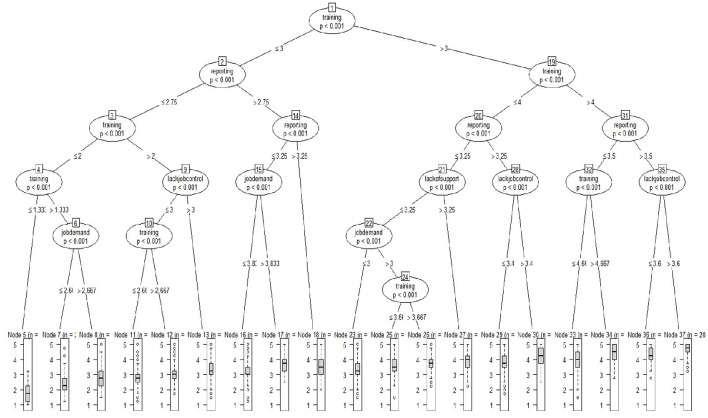
**Regression tree predicting perceived organizational support**. All variables in tree are positively coded.

The original article has been updated.

## Conflict of interest statement

The authors declare that the research was conducted in the absence of any commercial or financial relationships that could be construed as a potential conflict of interest.

